# General practitioners’ views on (long-term) prescription and use of problematic and potentially inappropriate medication for oldest-old patients—A qualitative interview study with GPs (CIM-TRIAD study)

**DOI:** 10.1186/s12875-017-0595-3

**Published:** 2017-02-17

**Authors:** Nadine Janis Pohontsch, Kathrin Heser, Antje Löffler, Britta Haenisch, Debora Parker, Tobias Luck, Steffi G. Riedel-Heller, Wolfgang Maier, Frank Jessen, Martin Scherer

**Affiliations:** 1 Department of General Practice/Primary Care, University Medical Center Hamburg-Eppendorf, Martinistr. 52, 20246 Hamburg, Germany; 2 Department of Psychiatry and Psychotherapy, University of Bonn, Sigmund-Freud-Straße 25, 53105 Bonn, Germany; 3 Institute of Health, Brandenburg University of Technology, Großenhainer Straße 57, 01968 Senftenberg, Germany; 4 German Center for Neurodegenerative Diseases (DZNE), Kurt-Georg-Kiesinger-Allee 3, 53175 Bonn, Germany; 5 Institute of Social Medicine, Occupational Health and Public Health (ISAP), University of Leipzig, Philipp-Rosenthal-Str. 55, 04103 Leipzig, Germany; 6 Clinic and polyclinic for psychiatry and psychotherapy, University Medical Center Cologne, Kerpener Str. 62, 50937 Cologne, Germany

**Keywords:** Potentially inappropriate medication, Qualitative interviews, Oldest-old patients, PRISCUS list, General practice

## Abstract

**Background:**

Potentially inappropriate medication (PIM) is defined as medication with uncertain therapeutic effects and/or potential adverse drug reactions outweighing the clinical benefits. The prescription rate of PIM for oldest-old patients is high despite the existence of lists of PIM (e.g. the PRISCUS list) and efforts to raise awareness. This study aims at identifying general practitioners’ views on PIM and aspects affecting the (long-term) use of PIM.

**Methods:**

As part of the CIM-TRIAD study, we conducted semi-structured, qualitative interviews with 47 general practitioners, discussing 25 patients with and 22 without PIM (according to the PRISCUS list). The interview guideline included generic and patient-specific questions. Interviews were digitally recorded and transcribed verbatim. We content analyzed the interviews using deductive and inductive category development.

**Results:**

The majority of the general practitioners were not aware of the PRISCUS list. Agents deemed potentially inappropriate from the general practitioners’ point of view and the PRISCUS list are not completely superimposable. General practitioners named their criteria to identify appropriate medication for elderly patients (e.g. renal function, cognitive state) and emphasized the importance of monitoring.

We identified prescription- (e.g. benzodiazepines on alternative private prescription), medication- (e.g. subjective perception that PIM has no alternative), general practitioner- (e.g. general practitioner relies on specialists), patient- (e.g. “demanding high-user”, positive subjective benefit-risk-ratio) and system-related aspects (e.g. specialists lacking holistic view, interface problems) related to the (long term) use of PIM.

**Conclusions:**

While the PRISCUS list does not seem to play a decisive role in general practice, general practitioners are well aware of risks associated with PIM. Our study identifies some starting points for a safer handling of PIM, e.g. stronger dissemination of the PRISCUS list, better compensation of medication reviews, “positive lists”, adequate patient information, multifaceted interventions and improved communication between general practitioners and specialists.

**Electronic supplementary material:**

The online version of this article (doi:10.1186/s12875-017-0595-3) contains supplementary material, which is available to authorized users.

## Background

The demographic trend resulting in increasing proportions of elderly people in all industrialized countries is an often cited phenomenon. Prescribing for elderly patients is a complex process influenced by many health-, health care system-, individual- and society-related factors. The line between rigorous treatment of diseases and harm induced by medications is thin [1, 2]. Potentially inappropriate medication (PIM) is defined as medication with low benefit-risk-ratios, uncertain therapeutic effects and/or potential adverse drug reactions (ADR) outweighing the clinical benefits [3]. Notwithstanding the potential risks of PIM, one must not forget that ‘potentially’ does not equal ‘actually’ inappropriate for everyone [4]. Different lists of PIM for the elderly exist, including the PRISCUS list (PL, used in Germany, [5]), the Beers Criteria [6], the EU(7)-PIM list [7] and FORTA [8]. Despite the efforts to raise awareness and to reduce the prevalence of PIM, as well as the existing strategies and helpful tools to discontinue certain PIMs [9, 10], the prescription rate of PIM is still high [11–13].

The PRISCUS list is a empirically derived list of PIM for elderly patients in German-speaking countries including 83 medications from the following agent groups: analgesics, anti-inflammatory drugs; antiarrhythmic drugs; antibiotics; anticholinergic drugs; inhibitors of platelet aggregation; antidepressants; antiemetic drugs; antihypertensive agents and other cardiovascular drugs; neuroleptic drugs; ergotamine and its derivatives; laxatives; muscle relaxants; sedatives, hypnotic agents; anti-dementia drugs, vasodilators, circulation-promoting agents; and antiepileptic drugs. It was disseminated to all German physicians [5], but is not legally binding for physicians in Germany. General practitioners (GPs) are not the sole provider of ambulatory medical care for the elderly. They function as gatekeeper for specialized care and case manager integrating all health care measures, but Linder and colleagues found that the release of the PL had no influence on GPs’ prescription behavior [12].

Some qualitative research exists in which GPs’ views on PIM prescription and polypharmacy were examined (e.g. [14–16]). Voigt et al. [15] interviewed a small sample of German GPs on PIM and found for example limited knowledge regarding PIM, missing alternatives to PIM and bad experiences with changes of medication to be subjective reasons for PIM prescriptions. Anderson et al.’s [14] systematic review on PIM found intrinsic (e.g. problem awareness and self-efficacy to alter prescriptions) and extrinsic (e. g. feasibility of altering prescriptions in routine care) reasons for PIM prescriptions, but among the 21 studies included were only two studies from Germany dealing with hypnotics [17] and proton pump inhibitors [18], the latter not being on the PRISCUS list. Another meta-synthesis found the need to please the patient, feeling forced to prescribe, tension between prescribing experience and guidelines and prescriber fear to be causal factors of potentially inappropriate prescribing (PIP). Of seven studies on PIP included none was conducted in the German health care system [16].

With Voigt et al.’s study [15] being the only and a also small-sampled study interviewing German GPs about PIM (from the PRISCUS list), a comprehensive study of German GPs’ views on problematic medication/PIM, the PL, as well as on (long-term) prescriptions of PIM (according to the PL) is to our knowledge still missing. Therefore, in the present study, the following research questions were addressed:

1. What do German GPs think about PIM, problematic medication and the PL?
2. What affects (long-term) use of PIM/problematic medication from the GPs’ point of view?

## Methods

The CIM-TRIAD project (“Contextual background for chronic use of inappropriate medication at high age”) is a qualitative multicenter study. The project is comprised of qualitative semi-structured interviews with triads of GPs, patients and associates from the AgeCoDe-cohort (“German Study on Ageing, Cognition and Dementia in Primary Care Patients”, e.g. [19]) in Hamburg (HH), Bonn (BN) and Leipzig (L). The AgeCoDe study is a prospective longitudinal study on the epidemiology of mild cognitive impairment and dementia in primary care patients over 75 years. This paper reports results from the GP-interviews of the CIM-TRIAD study. The study was reviewed and approved by the ethics committee of the Hamburg Medical Association (October 8th 2014, MC-251/14), the ethics committee of the University Hospital of Bonn (July 7th 2014, 169/14) and the ethics committee of the University of Leipzig (August 27th 2014, 269-14-25082014), and funded by the German Federal Ministry of Education and Research in Germany (grant numbers 01GY1311A-C).

### Participants and recruitment

We aimed at interviewing GPs treating patients from the AgeCoDe-cohort in HH, BN and L. Inclusion criteria for GPs were: 1) GP must treat an eligible patient and 2) the patient’s consent for the GP to be interviewed. The only exclusion criterion for GPs was the GPs’ unwillingness to be interviewed. Eligible patients were defined by age of 85 years and older, with long-term use of PIM according to the PL. For every included patient taking a PIM another patient of equal sex and comparable age never having taken a PIM (nonPIM) was included. The patients were identified by a pharmacist based on the medication use recorded within the AgeCoDe study. PIM-patients took at least one drug from the PL in the last two follow-up intervals available. Preference was given to cases who were taking drugs from the PL continuously for as many follow-up intervals as possible, including baseline. NonPIM patients did not take any drug from the PL in baseline and any follow-up interval. Eligible patients from the AgeCoDe-cohort, which consented to being contacted for other studies, were contacted by telephone by AgeCoDe-staff and asked to participate in our study. We obtained the patients’ written informed consent to contact the patients’ GP and to interview the GP about the patient. All interviewees gave their written, informed consent to be interviewed and for the interview to be digitally recorded, transcribed and used for the study.

### Sample

We conducted interviews with 42 GPs (female = 20, male = 22) discussing a total of 47 patient (some GPs were interviewed more than once because they treated more than one patient, those multiple interviews with the same GP were counted as one interview), 2 pilot interviews (to test and refine the topic guide) and 3 additional interviews without discussing a specific patient with male GPs. We decided to analyze the pilot interviews as well, as they only caused minor changes in the interview guideline. We discussed 25 patients with PIM (female = 21, male = 4, aged 86–94) and 22 nonPIM-patients (female = 19, male = 3, aged 86–96). Mean age of patients was 89 years in both groups. For further details on the patients see ([20], dealing with the patient interviews). 19 interviews took place in HH, 15 in BN and 13 in L. GPs were interviewed at the GPs’ offices (*N* = 40), at NJP’s office (*N* = 2) or by phone (*N* = 5, considered acceptable for expert interviews [21]). GPs had known the patients for anywhere between 3 months and about 30 years. Interviews lasted 17–89 min (Ø 48 min). For an overview of the PIM-agent groups discussed see Table 1.

**Table 1:** Agent groups taken by patients

Agent group according to the PRISCUS-list	Number of patients taking a PIM from this agent group^a^
1. Analgesics, antiphlogistics	2
2. Antiarrhythmics	3
3. Antibiotics	1
4. Anticholinergics	1
5. Antidepressants	1
6. Antihypertensives, cardiovascular drugs	2
7. Sedatives, hypnotic drugs	5/3^b^
8. Anti-dementia drugs, vasodilators, circulation-enhancing drugs	2
Agents from different agent groups (combinations of 7/8; 2/5; 1/8; 5/7 and 7/antiemetic drug)^c^	5
neuroleptics, ergotamine/ergotamine derivates, laxatives, muscle relaxants, antiepileptic drugs and anticoagulants/antiplatelet drugs	0

^a^Some patients took more than one PIM from the same agent group

^b^Number of patients taking more than one PIM from one agent group

^c^Numbers indicate agent groups taken by those patients

### Interview guideline

The semi-structured interview guideline [22] was developed by NJP and MS and was discussed and refined by the interdisciplinary CIM-TRIAD-study group (areas of expertise represented: psychology, medicine, psychiatry, public health and pharmacology). It was piloted in two interviews with practicing GPs and only slightly adapted afterwards. The interview guideline (see Additional file 1) consisted of generic questions concerning GPs’ views on problematic (“potentially inappropriate” from the interviewed GPs’ point of view, but not necessarily according to the PL; further definition below)/potentially inappropriate medications for the elderly, patient wishes, cooperation with pharmacies and information resources, and specific questions related to the patients (potentially inappropriate) medication (reasons for prescription, cessation tries, communication).

### Problematic medication

The PL is a tool based on empirical evidence and expert consensus [5]. Although they can be helpful tools, lists of PIM are considered controversial. Which drugs should (not) be included in these lists is intensively discussed [23, 24] and differs from country to country [5–8]. As there is no final consensus as to which medications need to be included in those lists and the content of lists being subjected to change we deem research on PIM not only from the view of the authors of the PL and other lists, but also from the users’ (GPs) point of view as highly necessary. Therefore we use the term problematic medication to define medication that is “potentially inappropriate” for the oldest-old patients from the individual GP’s point of view, but not necessarily according to the PL.

### Data collection and transcription

All interviews were conducted using the interview guideline which allowed the interviewer to ask individualized questions deviating from the pre-formulated questions in order to explore new or unexpected themes brought up by the interviewee. All interviews were conducted between December 2014 and July 2015 by AL (Master of Public Health), KH and NJP (both trained psychologists/postdoctoral researchers). If a GP treated more than one eligible patient in the respective study center, he or she was asked the generic questions only once but every patient was discussed separately. We digitally recorded the interviews which were then transcribed verbatim by a trained research assistant, who de-identified all interviewee and patient data during transcription. The accuracy of transcripts was checked by the respective interviewer.

### Data analysis

The transcripts were analyzed using structuring content analyses [25–27], using a combination of the directed approach (inductive coding) and the conventional approach (deductive coding). The transcripts were read several times before coding and abstracts were written in order to condense the most important information. The coding was conducted using the unabbreviated transcripts. Deductive categories on the highest level were derived from the research questions (see above, 1. views on PIM/problematic medication and the PL; 2. aspects affecting (long-term) use of PIM/problematic medication). Aspects affecting (long-term) use of PIM/problematic medication were grouped as being either prescription- and medication-, GP-, patient- or system-related and sub-codes were developed based on the existing research literature on PIM (e.g. [4, 9, 11, 14, 28, 29]). Deductive code development was conducted by KH, AL and NJP. These categories were supplemented by inductive categories formed during the material reviews by NJP in close consultation with MS (certified primary care physician, university professor). Due to the exploratory nature of the study and in order to make sure that not only pre-existing concepts are reflected in the categories [25, 27], the main focus was placed on inductive category formation. Deductive and inductive categories were described in code memos. To secure intersubjective comprehensibility and credibility [21] of the analysis the results were discussed in two face-to-face meetings of the CIM-TRIAD-study group and in a meeting of an interdisciplinary work group for qualitative methods in HH (led by NJP). Data was managed using MAXQDA 11 (Verbi GmbH).

## Results

**Fig. 1:**
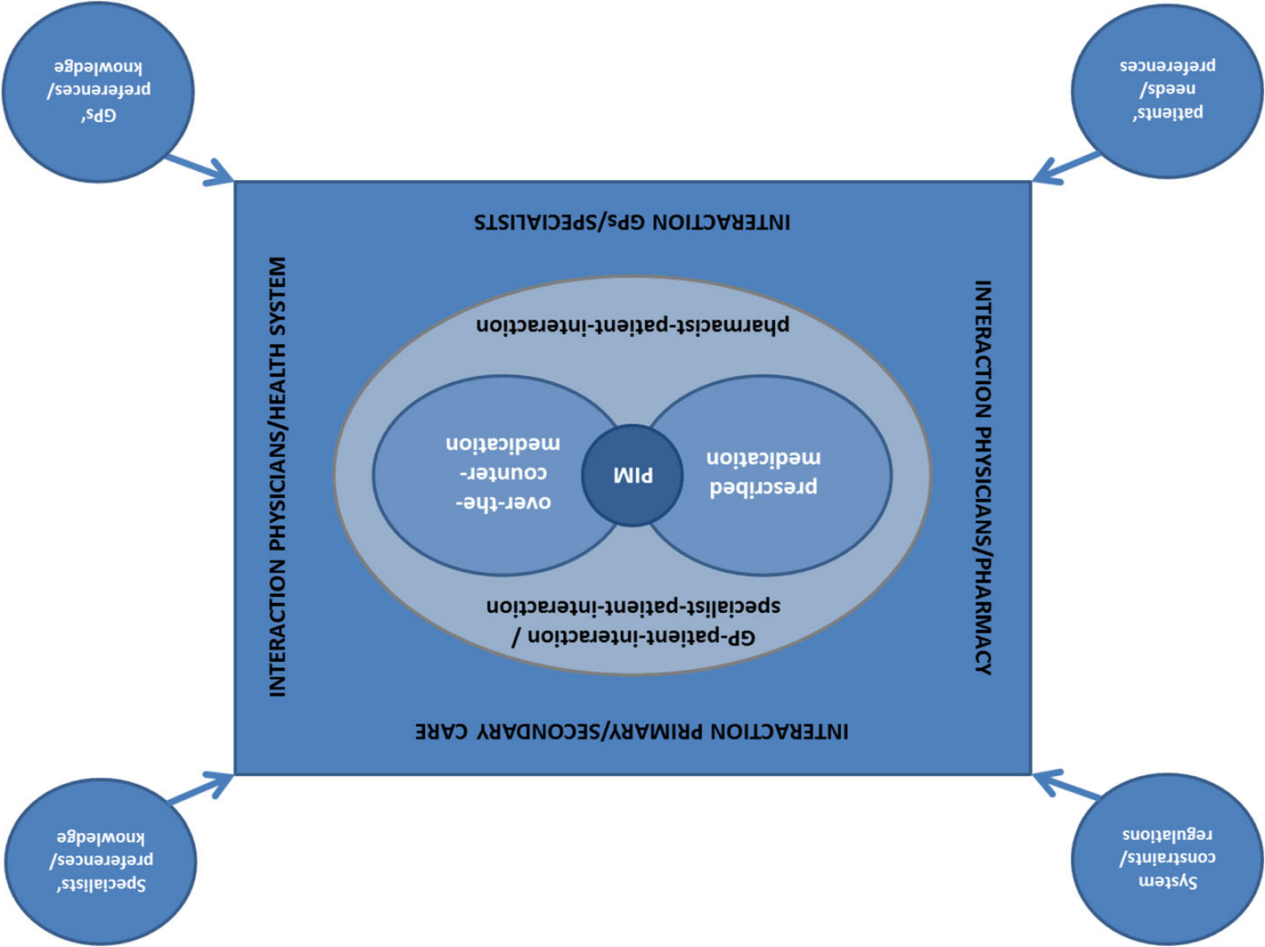
Context of potentially inappropriate medication

The results section is structured according to the research questions mentioned above. GPs’ general views on the PRISCUS list and problematic medication are followed by different aspects of (long-term) use of PIM and other problematic medications. Figure 1 shows an overview of the stakeholders involved, the interaction processes and the influence factors identified during the interviews.

Interviewee identifiers are structured to indicate that the interviewee is a GP (=HA), to identify the GP’s city of residence (L = Leipzig, HH = Hamburg, B = Bonn), to indicate whether the discussed patient was taking a PIM or not (PIM vs. nonPIM), and to indicate the patient number, that the interview was a pilot interview (e. g. Pt2) or had no patient relation (e.g. noPat1).

### PRISCUS list and problematic medication

The majority of GPs were not aware of the PL. GPs aware of the PL had mixed feelings about it. While some GPs appreciated the list, others had a more negative view, because they felt (severely) restricted in their freedom to choose medications. Rather than having a blacklist “banning” certain medications, they would prefer a whitelist indicating which medications can be safely used for elderly patients.

*“The PRISCUS list is a pain I’ll say. […], if I were to adhere to the PRISCUS list, I wouldn’t be able to prescribe them a single pill. […] I simply find it better to have concrete recommendations made for the elderly. […] I would prefer something with a positive formulation.”*

*(paragraph 38–44, HA_L_PIM09)*

Some agents on the PL were viewed as rather unproblematic, while others were missed on the list. Medication stated to be problematic by GPs [e.g. (oral) anticoagulants, non-steroidal anti-inflammatory drugs (NSAIDs)/opioids, sedating agents/antidepressants, and diuretics] did not intersect extensively with the PL.

*“[…] Surely some are unjustly on the list because the experts who created the PIM list were no practitioners […] I say this a bit degradingly: The list is politically correct. Everything is on it that should be on it.”*

*(paragraph 30, HA_HH_Pt2)*

Nevertheless, GPs mentioned criteria for the suitability of medications for the elderly which coincided with the PIM criteria (e.g. renal function, cognitive state, and benefit-risk-ratio) and strongly supported the significance of (long-term) monitoring for all kinds of side-effects in elderly patients.

*“[…] when the kidney function slowly decreases problems can come up, or when the liver function is reduced, […]. That’s why I do bloodwork regularly. […]”*

*(paragraph 41–43, HA_HH_nonPIM01)*

GPs described being aware that some of their prescriptions might be seen as potentially inappropriate. They justify the use of these medications with monitoring (e. g. blood tests, asking for side effects, ECGs).

*“[…], the medication [Acetyldigoxin is defined as PIM according to the PL] is actually used to, to reduce or regulate, to normalize the heartrate. […] the other organs function fine, in this case particularly if the kidney function is good, then the active metabolites can be excreted and there is no accumulation, which in turn wouldn’t lead to other side-effects. In this case, with Mrs. K, everything is in balance and (.) under control and without risks. […], in her case, too, it is a safe therapy, even though it has a negative connotation.”*

*(paragraph 152–157, HA_BN_PIM05)*

In addition to specific drugs/agent groups, polypharmacy was seen as the main risk factor for patient’s safety regarding their medication. Most GPs laid strong emphasis on the fact that they generally tried to reduce/prevent polypharmacy in their elderly patients. Reported reasons for polypharmacy were specialist consultations and hospital stays leading to an uncritical application of treatment recommendations from different disease-specific guidelines.

*“Well, when I started out, I always said three medications are enough. There generally aren’t any more. […] when they come out of the hospital categorized […] according to their CHADS-scores and guidelines, one has quite a time of it because God knows they have way more than three medications, you know?”*

*(paragraph 11, HA_HH_nonPIM07)*

### Aspects of (long-term) use of PIM and other problematic medication

According to GPs, there is a high diversity of agents and many problematic medications either missing on the PL or listed despite being viewed as rather unproblematic (see above). Therefore, we will depict the most important prescription- and medication-, GP-, patient- and system-related aspects of (long-term) use of PIM and medication deemed problematic by GPs.

#### Prescription- and medication-related aspects

The prescription of benzodiazepines and z-drugs is restricted to a maximum of 4 weeks by drug regulating authorities in Germany. The prescriptions are also controlled by health insurance funds. The interviewed GPs know the regulations and risks of those substances and declared to be very reluctant to start this kind of medication. Either way, they often choose to bypass the control mechanisms with alternative private prescriptions for older patients with a long-term, low-dose-dependency. They declared that this strategy is used to keep the medication dose which patients take low via financial incentives.

*“ […] that it is written on a private prescription. […], that it isn’t followed up on then. Otherwise there are very clear rules from the KV [Association of Statutory Health Insurance Physicians], one isn’t allowed to prescribe them longer than 4 weeks with very few exceptions. Some then receive a private prescription. […] The good thing about the private prescription is (.) that they actually use less. […]”*

*(paragraph 26, HA_HH_PIM02)*

The non-use or discontinuation of PIM/problematic medication is sometimes hindered by the subjective perception that alternatives are less potent (especially for sleep-inducing drugs/pain killers). If the PIM is the only drug with a positive effect, it might be continued despite the potential risks.

*“[…], that a medication, […] is potentially problematic for a patient or has significant side-effects, then you may have to restrict the medication to the available alternatives, which are not always equal in their effect, you know? […]”*

*(paragraph 78–80, HA_BN_PIM09)*

Often, the potential negative effects attributed to the drug never occur (even with long-term use). GPs tend to resign from cessation of potentially inappropriate long-term medication when they do not register any ADR.

*“Well, certain blood pressure medications, […] like Doxazosin [Doxazosin is defined as PIM by the PL] […], some elderly patients still have in their medication. […] Because they’ve had it for 20 years and, thus far, everything went well. […], if they say, […] they feel well, then I leave it with them. […]”*

*(paragraph 37–39, HA_L_noPat1)*

#### GP-related aspects

GPs criticize unnecessarily extensive or inappropriate medication plans established by specialists and in hospitals, but also report a trust in specialists’ prescriptions. This apparent contradiction is resolved by the fact that criticism is often related to medications also prescribed by general practitioners (e. g. antihypertensives, analgesics) and not to medication prescribed solely by specialists (e.g. cancer or hepatitis treatment, urologic antibiotics). Drugs prescribed by specialists are less prone to be questioned after referrals.

*“When I send someone to a specialist, then I want to make use of that specialist’s expertise, it would be absurd if I would say “That’s nice, but we aren’t doing any of that.”. […]”*

*(paragraph 89, HA_HH_nonPIM05)*

GPs do see specialists as responsible for monitoring and controlling risks and benefits of their prescriptions. GPs reported that this monitoring does not seem to happen regularly and specialists do not reflect about (long-term) usefulness and risks of their prescriptions. GPs were not willing to take responsibility for specialists’ prescriptions, but often have no choice but to do so anyway.

*“Except for the incontinence medication [Solifenacin is defined as a PIM according to the PL], she gets all her prescriptions from me, yes. There are certain specialists who I don’t want to exclude/from their responsibilities. […] that the patients remain in contact with the treating specialists and can give direct feedback […]. And I don’t want to take on the responsibilities of all specialists by myself; I want to keep them in the boat. […]”*

*(paragraph 126, HA_HH_PIM10)*

GPs showed a high awareness of the risks of problematic medication/PIM and polypharmacy. Most of them stated that keeping a patient’s number of medication as low as possible was their highest priority.

*“[…] Well the reduction of medications is certainly a high priority, in my opinion a GP’s greatest skill, taking away medications.”*

*(paragraph 11, HA_L_nonPIM08)*

In doing so, GPs trust in a small spectrum of medications, whose risks and benefits are well-known and calculable to them. Having to treat illnesses/diseases from many different specialties, this is their initial strategy for risk reduction.

*“[…], for every type of illness, let’s say, one uses particular substances and always the same ones and one knows them well and doesn’t go along with every new medication trend. I, for one, tend to use medications, which have long since been tried and tested […].”*

*(paragraph 85, HH_HA_nonPIM01)*

#### Patient-related aspects

Some patients with many/very strong afflictions tend to be very open-minded towards polypharmacy. From the GPs’ point of view they act demanding and call on different physicians for the same medical conditions to get medication. GPs report these demanding high-users to be at a higher risk of getting lots of prescriptions because prescribing is one approach of handling this behavior on a short term basis.

*“And he will always give a plausible explanation as to why he still needs the [sleep-inducing drug] or still needs it for a while, you know? […] And sometimes it is easier to just fulfill the wish and say “OK, for God’s sake,” than to say “I won’t do it anymore.”.”*

*(paragraph 92–96, HA_BN_PIM09)*

The high demand established by these patients may lead to treatment errors when relevant context factors were not born in mind (e.g. other treating physicians, medication prescribed by other specialists).

*“[…] some patients are very demanding; Mrs. S is also very, very demanding. […], they want a pill for every little thing. […] But Mrs. S is someone, who simply wants everything, everything that is available. […]”*

*(paragraph 9, HA_L_nonPIM06)*

Sometimes the multi-morbid patient’s distress, as perceived by the GP, is so high that this entices the GP to prescribe problematic medication/PIM.

*“[…] Well, she has a tinnitus, which […] keeps her from sleeping. That is also the reason why she became addicted to sleep inducing medications. […] Oh, tried with all alternatives, to somehow solve the problem in another way. […] It is such a vicious cycle, where she says “If I cannot sleep, my blood pressure goes through the roof, I get totally nervous, my depression gets worse when I don’t sleep.” In the end, the sleep-inducing medication is the lesser of two evils. […]”*

*(paragraph 468, HA_BN_PIM02)*

Two agent groups exist — sleep-inducing drugs and NSAIDs — in which the patients feel that the subjective (and immediately perceptible) benefit outweigh the known, but not yet experienced, risks. This leads to a constant demand for sleep-inducing drugs and intake of over-the-counter NSAIDs.

*“[…] “Oh, I’m already so old now, what does it matter now.” Right? And when I tell them that they can become dependent on [sleep-inducing medications] and that they then won’t sleep more but increase their risk of falling and reduce their cognitive abilities. “Oh well, what does that matter now?”. […]”*

*(paragraph 8, HA_HH_PIM02)*

GPs also reported communication deficits with elderly patients. There are instances where elderly patients consult specialists without having consulted the GP first, do not report an intake of over-the-counter medications (considering them to be harmless) or experienced side effects, and cannot give adequate information about their medication regimen or exams performed by specialists.

*“ Especially the co-medications which are available over the counter without a prescription are extreme amongst sleep-inducing medications and pain-killers. […] One has to actively ask about them, because the patients generally don’t consider these medications. […] They rather think “Well if I can buy them like that they cannot be that bad.”.”*

*(paragraph 107–113, HA_BN_PIM02)*

*“Especially with the new oral anticoagulants, […]. In the practice we ask “Do you take Marcumar or Aspirin?” and they say “No.”, because it has a different name. And just like that you’ve given an injection or a medication which can cause complications.”*

*(paragraph 58, HA_HH_PIM06)*

#### System-related aspects

GPs often stated that patients have several treating physicians, who do not adequately communicate with each other. GPs often do not receive specialists’ letters or discharge documents, do not know which specialists the patients have seen and which medications were prescribed by the specialists.

*“[…] If he goes to the urologist and to this and that specialist with his chip card and without my knowledge, I don’t get a report and I don’t know what all was prescribed. […] The orthopaedist never, doesn’t write a report. Urologist X, right around the corner from the patient, never does. The otolaryngologist is Mr. Y, he doesn’t either. […]”*

*(paragraph 31, HA_HH_nonPIM06)*

GPs also often criticize specialists’ lack of a holistic or geriatric view on elderly patients. Compared with the GP, they know much less about the patients concerning comorbidities, established medications or other specifics (e.g. medication sensitivity, changed metabolism) and may, therefore, consider risks and benefits less.

*“[…] A classic is of course Ibuprofen. Well, Diclofenac, NSAIDs which are taken very, very often. […] I always try to include the orthopaedist, […] they very, very quickly recommend […] this group [of medications] without asking themselves, “Is there a pre-existing internal condition?”. […]”*

*(paragraph 42, HA_HH_PIM08)*

This might lead to a different priority setting, polypharmacy, the prescription of problematic medications/PIMs and unforeseen drug-drug-interactions.

*“And the more physicians are consulted, the more is added. Seldom subtracted, right?”*

*(paragraph 24, HA_BN_nonPIM06)*

The coordination of treatment, monitoring and (dis-)continuation of drugs is seen as an essential duty of GPs, who (shall) function as gate keeper and case manager in the German health care system. Although GPs tend to trust in specialists’ prescriptions (see above), there seem to be instances in which GPs change or discontinue specialists’ medications they deem as problematic. This seems especially true for drugs from the spectrum of internal medicine (e.g. heart and diabetes medication, painkillers). Yet a prerequisite for this is that the GPs know of all medications prescribed by other physicians and taken by the patient.

*“If I see an elderly lady/[…] whose bloodwork etc. I have due to regular consultations, unlike colleagues, (.) and specialized colleagues, and I know, for example, about […] the kidney function or the heart attack in the history etc., then […] they come back from the orthopaedist and have their painkillers in their bag, and I immediately discontinue their use. Rigorously. (..) And then they receive a different (.) painkiller from me.”*

*(paragraph 42, HA_HH_PIM05)*

Another point hindering the profound discourse over and review of medications taken by a patient is that there is often a dominating reason for the consultation. In this case, acute treatment needs let medication reviews slip through the net. If the patient does not report ADRs, a critical review of their medication might be forgotten or not seen as relevant at the moment.

*“[…] if the patients are well informed about what, yes, what’s important and what use and risks are behind it, then one can often make a good, mutual decision […] OK, I’ll say one doesn’t do that with every patient in daily practice, who, well not during every consultation, you know?[…], of course even now you don’t start from scratch, rather, if you see that things are going well and the patient feels well, then one doesn’t change anything. […]”*

*(paragraph 66–68, HA_BN_nonPIM05)*

## Discussion

### Main findings

The majority of GPs were not aware of the PL. Drugs deemed potentially inappropriate from the GPs’ point of view and the PL are not completely superimposable. GPs have lots of criteria to identify (in-)appropriate medication for elderly patients which are similar to those underlying the PL. They emphasized the importance of constant monitoring.

We identified prescription- (e.g. benzodiazepine on an alternative private prescription), medication- (e.g. subjective perception that a PIM has no alternative), GP- (e.g. GP relies on specialists), patient- (e.g. “demanding high-user”, subjective benefit-risk-ratio) and system-related aspects (e.g. specialists lacking holistic view, interface problems between treating physicians) of (long-term) use of PIM/problematic medication.

### Strengths and limitations

To our knowledge this is the first qualitative study asking a large number of GPs from different study centers about their views on problematic medication, on the PL and on (long-term) use of PIMs, as well as discussing specific cases of (long-term) use of PIMs according to the PL. Thereby, individual-, system- and medication-related aspects of (long-term) use of PIM/problematic medication were identified.

We included GPs depending on the PIM status of one of their patients. This allows no conclusions about the GPs’ tendency to treat their patients with(out) PIM. GPs recruited for treating a patient with PIM might not be different from those recruited with a nonPIM-patient regarding their likelihood to treat a patient with prescriptions from the PL. In some cases the GPs did not prescribe the PIM taken by their patients (over-the-counter PIM or PIM prescribed by a specialist). Anyway, we felt that GPs are highly aware of the potential inappropriateness of many medications for elderly patients, even if the medications named were not always the same as listed in the PL.

The PIM-patients mostly received long-term prescriptions of PIM. A repeated prescription of a PIM could be understood as an indicator that the medication was tolerated by the patient [28]. A PIM might have been discontinued after short-term use in patients with stronger ADRs or patients might be deceased due to ADR, which both would have resulted in not being included in our study. The majority of patients were female, which is in line with findings from other studies that women are at higher risk to get a potentially inappropriate prescription than men [28]. We also did not include patients without associates or patients with major cognitive deficits. It cannot be ruled out that a different sample of patients would have triggered different accounts from the GPs.

### Discussion of results and comparison with existing literature

Our findings are in general consistent with and do not oppose findings in the literature known to us (for example [4, 14–16, 30], but offer new insights on the prescription of PIMs too. The PL is little known and even less used in our interviewed population, which is in line with findings from other qualitative studies on the Beers criteria [4] and the PL [15]. With the PL not being legally binding in the German health care system, this finding is not surprising as it resembles the problems of dissemination and use of other medical guidelines [31, 32] and tools to help appropriate prescribing [10, 33].

The interviewed GPs tried to avoid polypharmacy wherever possible and use only well-known and well-tried medications. GPs interviewed on potentially inappropriate prescribing by Clyne et al. [30] also stated that multimorbidity and polypharmacy contribute to the high complexity of treating elderly patients. One could say that our interviewees tend to use individual “whitelists” and wish for official positive lists (of medications to prescribe). The PL includes possible therapeutic alternatives for PIM and could fulfill this need if known to the GPs. Efforts have been made to create consensus lists of essential and useful medications for general practitioners in other contexts as well [34, 35]. Our results show a need for making the PL and the like known to GPs more widely. But one should keep in mind that all kinds of lists can only support clinical decision making and might clash with actual treatment reality in individual cases. For example GPs reported—despite being aware of the potential harmfulness of some medication—to have no potent alternatives for the PIM and justify the use of these medications with constant monitoring and the non-occurrence of side effects. Other studies report similar accounts on unavailability of alternatives [15, 16] and monitoring [15]. Voigt et al.’s [15] results also support our finding that a patient’s distress might sometimes justify the use of PIM. Anderson et al. [14] and Cullinan et al. [16] also found that rating a drug as potentially harmful in general did not always keep GPs from prescribing it to an individual patient and that the drug appearing to work without side effects perpetuates the prescription.

Spinewine et al. [36] report that the focus on and considerable time needed to treat acute problems in acute wards for the elderly leads to other prescriptions (for chronic problems) being overlooked and that the transfer of information between primary and secondary care is often limited (see also [14]). Our interviewees gave accounts of a similar phenomenon in treating elderly patients in primary care. Acute problems dominate the short available consultation time leading to omitting regular medication reviews and discharge documents from hospitals are often not received.

Adherence to (different) guidelines leads to polypharmacy in multi-morbid patients. This increases the risk of PIM especially for elderly people with comorbidities, which are not sufficiently represented in such specialized guidelines [37]. This implies the need for a guideline on how to handle multi-morbid (elderly) patients, as it was recently published by the National Institute for Health and Care Excellence [38] and is currently under construction by the German College of General Practitioners and Family Physicians [39].

The PL is—like the Beers criteria or FORTA—an awareness raising tool derived from empirical evidence and expert consensus [5, 6, 8]. Nonetheless, these lists are considered controversial as there is considerable disagreement about which drugs should be on these lists and whether indications/diagnoses should be included or not [23, 24]. Results concerning adverse outcomes are inconsistent [40]. Most ADRs and hospitalizations due to ADRs might not be related to PIM, but to a few commonly used drugs (e.g. warfarin or insulin, [41]). Our findings show that GPs’ and PL’s assessments of potential inappropriateness are not superimposable and that the prescription of PIM or problematic medication can be legitimatized by constant monitoring and non-occurrence of ADRs. As mentioned above other research supports these findings [14, 15, 42]. Medication classification systems might be at odds with the complex and multidimensional considerations needed in medication decision making of GPs [4].

The Medicinal Products Directive recommends prescribing benzodiazepines and z-drugs for no longer than 4 weeks [43]. Bypassing the statutory health insurance control mechanism for monitoring prescriptions of these drugs seems to be common in Germany, the relative risks of a patient receiving an out-of-pocket, private prescription is higher than for any other medication [44]. Jahnsen et al. state a lack of knowledge concerning reasons for long-term prescription of these drugs [45]. In our study reasons for bypassing the control mechanisms were the non-occurrence of ADR, the feeling that alternatives were much less potent, a subjective positive risk-benefit-ratio from the patient’s point of view and a long-term low-dose dependency (making the patient demand more prescriptions). Other studies also show that the risks of benzodiazepine use are usually perceived as low by patients [29]. Sometimes patients even diminish side-effects of benzodiazepines [46]. GPs mentioned that trying to discontinue psychoactive drugs, especially benzodiazepines, leads to patients switching to another GP who is more willing to prescribe these drugs [32]. Other GPs from Germany [15, 17] and other countries [14, 16] reported the same concerns. Another study by Dalleur et al. [33] supported that GPs fear reluctance of patients to change their treatment as there might be a high psychological or physical attachment to certain drugs deemed potentially inappropriate.

Based on our results it might not be enough to educate just the prescribers, especially because some PIM from the PL are over-the-counter-medications. Also, German patients have a free choice of medical practitioners allowing them to switch physicians when they are not satisfied with one’s prescription behavior (e.g. denying benzodiazepines). Despite patients’ complains about the (high) number of medications they have to take, our results show that they may be reluctant to cease taking medications. Patient-centered interventions are less common and sometimes even useless in reducing the number of medications [47]. Therefore, interventions should be individualized and must involve different healthcare professionals (pharmacists, nurses and GPs [48, 49]). Advancing regular medication reviews (prefixed by pharmacists and prepared by nurses) and adequate compensation and development of measures/materials to inform patients about potentially harmful effects of the most prominent PIMs (without frightening or overloading them with information [50]), might help in reducing the intake of PIM even though patients tend to trust their GPs more than patient information leaflets [48].

The existence of interface problems and communication barriers between specialists, physicians in hospitals, general practitioners and their patients is a well-known, intensively discussed fact. Treatment reports from specialists and discharge letters from hospitals were reported as often not reaching the GP and patients as (un-)intentionally not disclosing their full medication records by our interviewees and in different other studies [14, 30] too. ‘Fragmentation of care’ and involvement of more than one prescriber increase the number of medications on a patient’s list [30]. Hospitalizations may increase the number of PIMs [51] too. Our research and other studies show that GPs sometimes do not dare to change/discontinue medications prescribed by a specialized physician in a hospital [37, 52] or in the ambulatory setting [14, 16]. Establishing better communication routes/routines between GPs and specialists could help the GP to monitor patients for possible adverse drug reactions and harmful drug-drug-interactions. Electronic health cards, as means to record emergency data, prescriptions and medication plans [53] could be helpful, but still raise concerns about data safety and confidentiality [54].

## Conclusions

Lists of PIM are not uncontroversial [40, 55] and it still needs to be definitely proven that drugs deemed to be potentially inappropriate for elderly patients, really have worse benefit-risk-ratios and are responsible for more ADRs than other drugs [56]. Many lists of PIMs, interventions aimed at reducing polymedication, drug-drug-interactions, ADRs and tools to support physicians’ (de-)prescription exist, but do not seem to have reached the intended goals yet. The answer to the problem does not seem to lie in mono-causal pharmaco-centered approaches or practical helps/tools. Nonetheless, our results suggest areas which can be worked on to reduce those medications with a high potential to harm the relatively large group of elderly patients [57]. Based on our findings, strategies involving all stakeholders of the medication process: patients, general practitioners, specialists, physicians in hospitals, pharmacists in and out of hospitals, nurses (in hospitals), medical assistants as well as informal and formal caregivers (nursing services, nurses in nursing homes) etc.; should be implemented.

## Additional file

Additional file 1: Interview guideline. Interview guideline used for the semi-structured interviews in the study. (DOC 77 kb)

## Abbreviations

ADR: Adverse drug reactions
AgeCoDe: German Study on Ageing, Cognition and Dementia in Primary Care Patients
B: Bonn
CIM-TRIAD: Contextual background for chronic use of inappropriate medication at high age
DEGAM: German College of General Practitioners and Family Physicians
GP: General practitioner
HH: Hamburg
L: Leipzig
nonPIM: Without potentially inappropriate medication
NSAID: Non-steroidal anti-inflammatory drug
PIM: Potentially inappropriate medication
PIP: Potentially inappropriate prescribing
PL: PRISCUS list
