# Supplementary material for: General practitioners’ views on (long-term) prescription and use of problematic and potentially inappropriate medication for oldest-old patients—A qualitative interview study with GPs (CIM-TRIAD study)
Source: BMC Fam Pract. 2017 Feb 17;18:22. doi: 10.1186/s12875-017-0595-3 (PMC5395870; doi:10.1186/s12875-017-0595-3)
Supplement: Additional file 1: — Interview guideline. Interview guideline used for the semi-structured interviews in the study. (DOC 77 kb) [file 12875_2017_595_MOESM1_ESM.doc]

Additional file: Interview guideline

| What do you think about problematic medications in elderly patients? |
| --- |
| How do you handle problematic medication in your elderly patients? |
| Please describe a situation where a problematic medication played a decisive role for an elderly patient. For example, where undesired side effects or conditions occurred or where the need to discontinue the medication came up. |
| How do you evaluate whether or not a drug is appropriate for a specific patient? |
| Which medications do you deem inappropriate for elderly patients? Why? |
| Some medications require monitoring. How do you decide whether or not to prescribe such medications to an elderly patient? |
| How do you talk about the medications you prescribe with your elderly patients? |
| Do elderly patients sometimes approach you with certain notions about afflictions and symptoms for which you are then supposed to prescribe something? Which notions? How do you react? |
| Do elderly patients sometimes categorically decline certain medications? How do you react? |
| How do you inform yourself about new medications with which you haven’t come in contact? |
| There are different approaches to support physicians in their prescription behavior, e.g. guidelines, alerts, practice software and the PRISCUS list. Which do you consider to be helpful which writing prescriptions? Please give an example where one of these instruments has been helpful to you. |
| Are there other occasions where you inform yourself about medications? |
| How do you cooperate with pharmacies? Please describe a case where a pharmacy contacted you about a drug you had prescribed. |
| We have talked a lot about problematic medications in the elderly in general. Now I would like to talk to you about Mrs./Mr. XY. Could you describe your relationship with Mrs./Mr. XY? |
| Which medications does she take? For how long now? |
| Which of these medications did you prescribe? |
| Which medications were initially prescribed by another physician from the ambulatory sector or by a hospital? |
| Have there been any recent changes to the medication plan? Why were changes made? |
| (only for GPs treating a patient with PIM)  If the GP prescribed the PIM:  The medication XY is considered potentially inappropriate for the elderly population although there may be good reasons for prescribing it anyway. What motivated you to prescribe this drug despite its potential inappropriateness?  If another specialist prescribed the PIM:  The medication XY is considered potentially inappropriate for the elderly population although there may be good reasons for prescribing it anyway. What motivated you to continue prescribing this medication? |
| How did you speak with the patient about the advantages and disadvantages of the medication(s)? |
| Which side effects/complications occurred? What did you do? |
| Has discontinuing the medication been considered before? If so, why? What happened then? |
| Do you want to add something concerning problematic medications in elderly patients? |
